# *In vitro* gastrointestinal digestion promotes the protective effect of blackberry extract against acrylamide-induced oxidative stress

**DOI:** 10.1038/srep40514

**Published:** 2017-01-13

**Authors:** Wei Chen, Hongming Su, Yang Xu, Chao Jin

**Affiliations:** 1 Department of Food Science and Nutrition, Zhejiang Key Laboratory for Agro-Food Processing, Zhejiang University, Hangzhou 310058, China

## Abstract

Acrylamide (AA)-induced toxicity has been associated with accumulation of excessive reactive oxygen species. The present study was therefore undertaken to investigate the protective effect of blackberry digests produced after (BBD) *in vitro* gastrointestinal (GI) digestion against AA-induced oxidative damage. The results indicated that the BBD (0.5 mg/mL) pretreatment significantly suppressed AA-induced intracellular ROS generation (56.6 ± 2.9% of AA treatment), mitochondrial membrane potential (MMP) decrease (297 ± 18% of AA treatment) and glutathione (GSH) depletion (307 ± 23% of AA treatment), thereby ameliorating cytotoxicity. Furthermore, LC/MS/MS analysis identified eight phenolic compounds with high contents in BBD, including ellagic acid, ellagic acid pentoside, ellagic acid glucuronoside, methyl-ellagic acid pentoside, methyl-ellagic acid glucuronoside, cyanidin glucoside, gallic acid and galloyl esters, as primary active compounds responsible for antioxidant action. Collectively, our study uncovered that the protective effect of blackberry was reserved after gastrointestinal digestion in combating exogenous pollutant-induced oxidative stress.

Acrylamide (AA) is a well-known toxicant that has attracted increasing attention after an announcement by Swedish National Food Authority and the University of Stockholm, who jointly claimed the discovery of AA in a variety of carbohydrate-rich foods subjected to high heat during food processing^1^,^2^,^3^. It has been confirmed that two major ingredients: amino acid asparagine and reducing sugars from Maillard reaction are predominantly precursors to AA. AA was classified as ‘probably carcinogenic to humans’ by the International Agency for Research on Cancer (IARC) in 1994^4^. Previous studies showed that AA caused damage to nervous system if exposed to high levels^5^,^6^. AA was also considered as a toxin with mutagenic property^7^. Since dietary AA could be easily absorbed by the human body^8^, extensive studies were performed to figure out the underlying mechanism (s) of AA-induced toxicity. AA derived from food processing possessed potential neurotoxic, genotoxic, carcinogenic, developmental, and reproductive toxic effects both *in vivo* and *in vitro*^9^,^10^. Therefore, potential harm of AA on human health cannot be ignored.

Accumulating evidence indicated that AA exposure resulted in oxidative stress both in cells and tissues^11^. Oxidative stress reflects an imbalance between the systemic impairment triggered by reactive oxygen species (ROS) and a physiological ability to readily detoxify the harmful intermediates. The disturbed cellular redox condition subsequently led to cyto- and geno-toxicity^12^. Recently, increasing attention has been paid to naturally occurring antioxidants derived from vegetables and fruits for protection against AA-induced toxicity^13^,^14^. For example, allicin derived from garlic showed protective effect against AA-induced hepatocyte damage^14^. Our previous study also reported that hispidin and myricitrin displayed a prominent antioxidant activity against AA-induced oxidative stress in Caco-2 cells^15^,^16^. However, the relationship between AA-induced toxicity and cellular oxidative stress is still unclear.

Blackberry is well known to contain abundant polyphenols that contribute to its high antioxidant capacity^17^,^18^. A large number of studies have confirmed that blackberry possessed potent biological activity and may provide health benefits including anti-hyperglycemic, anti-obesity as well as anti-inflammatory effects^19^,^20^,^21^. It is noteworthy that food will be subjected to digestive conditions within the gastrointestinal tract. During digestion, not only antioxidants, but also many other functional components are immersed in digestive juices. As a consequence, the biological activities of functional components may be altered and some substances may be transformed into other compounds with varied bioactivity and bioavailability^22^. Although some studies have addressed the health benefits of phenolic compounds present in blackberry, studies concerning the effect of blackberry extract before and after GI digestion need to be elucidated. In the present study, an *in vitro* digestion model was employed to investigate the influence of simulated gastrointestinal digestion on the protective effect of blackberry against AA-induced oxidative damage.

## Results and Discussion

### Effect of blackberry digests (BBD) on AA-induced cytotoxicity and genotoxicity in HepG2 cells

**Figure 1:**
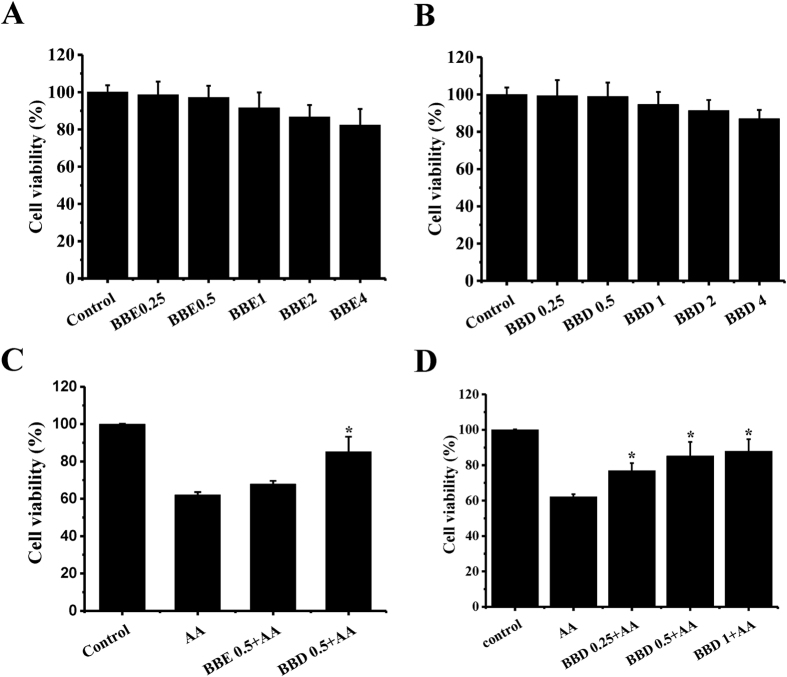
Effects of BBE and BBD on AA-induced cytotoxicity determined by MTT before and after *in vitro* digestion. (**A**) The influence of BBE (0.25, 0.5, 1, 2 and 4 mg/mL) on cytotoxicity in HepG2 cells. (**B**) The influence of and BBD (0.25, 0.5, 1, 2 and 4 mg/mL) on cytotoxicity in HepG2 cells. (**C**) Effect of BBE (0.5 mg/mL) or BBD (0.5 mg/mL) on AA induced cytotoxicity. HepG2 cells were exposed to 2.5 mM AA for 24 h in the presence or absence of BBE (0.5 mg/mL) or BBD (0.5 mg/mL) and then cell viability was determined by MTT assay. (**D**) The influence of BBD (0.25, 0.5 and 1 mg/mL) on cytotoxicity in HepG2 cells. The results were expressed as mean percent (means ± SD of three independent experiments). *P < 0.05 represents significant difference compared with AA treatment group. BBE: Blackberry extract; BBD: Blackberry digests.

To evaluate the protective role of blackberry extract produced before and after GI digestion on AA-induced cytotoxicity, HepG2 cells are employed for investigation. First, we tested whether BBE (0.25, 0.5, 1, 2 and 4 mg/mL) and BBD (0.25, 0.5, 1, 2 and 4 mg/mL) may cause toxicity in HepG2 cells. There was no toxicity observed in the treatment of either BBE or BBD (Fig. 1A,B). Next, we observed the cell viability, determined by MTT assay, markedly decreased to 62.1% in the presence of 2.5 mM AA for 24 h (Fig. 1C,D). To examine the protective effect of BBE and BBD on AA exposure, HepG2 cells were pretreated with 0.5 mg/mL of BBE or BBD for 2 h and then incubated with 2.5 mM AA for 24 h, followed by MTT assay. Pretreatment with BBD led to a significant increase in cell viability (85.2%) compared with that in AA-treated group. However, pretreatment with BBE caused a slight increase in cell viability (67.8%). Moreover, pretreatment of BBD within concentrations (ranging from 0.25, 0.5 to 1 mg/mL) contributed to an increase in cell viability (Fig. 1D).

**Figure 2:**
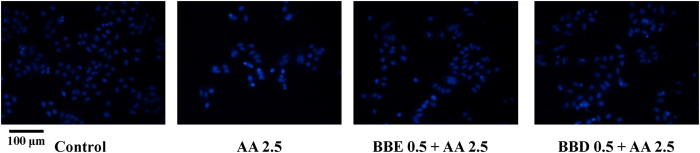
Effects of BBE and BBD on AA-induced genotoxicity determined by nuclear staining of HepG2 cells with Hoechst 33258. Cell morphological image of HepG2 cells. HepG2 cells were exposed to 2.5 mM AA for 24 h in the presence or absence of BBE (0.5 mg/mL) or BBD (0.5 mg/mL). *P < 0.05 represents significant difference compared with AA treatment group. BBE: Blackberry extract; BBD: Blackberry digests.

Based on this observation, we hypothesized that BBD might provide protection against AA-induced genotoxicity in HepG2 cells. To test this hypothesis, Hoechst 33258, a specifically blue fluorescent dye highly sensitive to genomic DNA, was used to detect the degree of nuclear damage. As shown in Fig. 2, exposure of 2.5 mM AA contributed to an increase of small bright blue dots representing chromatin condensation or nuclear fragmentation in HepG2 cells^23^. However, after pretreatment with BBD (0.5 mg/mL) or BBE (0.5 mg/mL), there was almost no small bright blue dots observed obviously, especially in BBD pretreatment, which indicated that pretreatment with BBD could effectively attenuate AA-induced nuclear damage in HepG2 cells. Collectively, these results suggest that the resultant blackberry digests produced from GI digestion could enhance the cell viability and mitigate genotoxicity induced by AA in HepG2 cells.

### BBD attenuated AA-induced ROS generation

**Figure 3:**
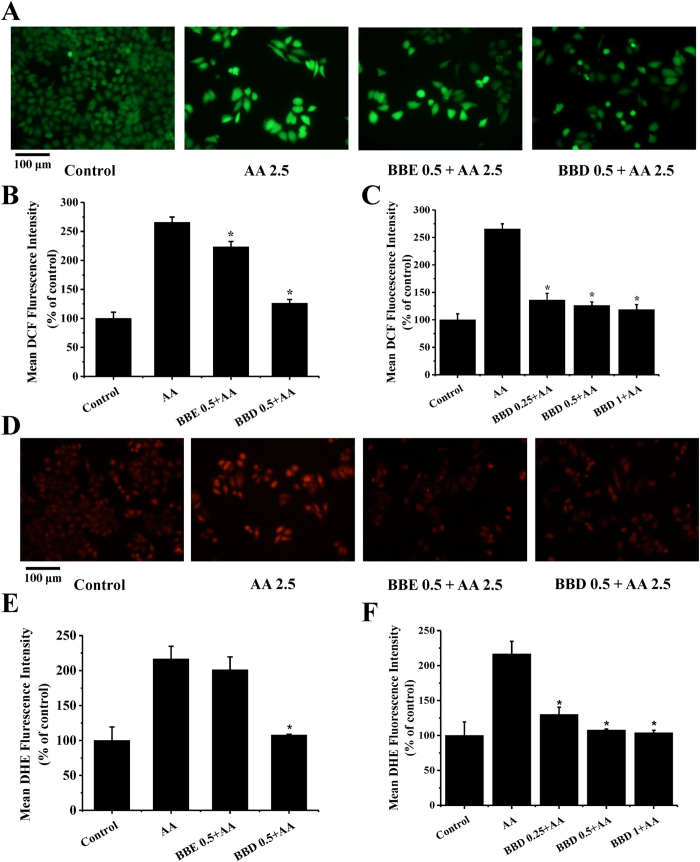
Effect of BBE and BBD on AA-induced ROS and superoxide anion radicals production in HepG2 cells. (**A**) Effect of BBE and BBD on AA-induced ROS. After treatment with 2.5 mM AA in the presence or absence of BBE (0.5 mg/mL) or BBD (0.5 mg/mL) for 24 h, cells were collected and incubated with 10 μM of DCF at 37 °C for 30 min, then cells were washed with PBS and evaluated by fluorescence microscope. (**B**) The quantitative data of panel (A) and results were expressed as mean DCF fluorescence intensity (means ± SD of three independent experiments, n = 6). (**C**) Effect of BBD (0.25, 0.5 and 1 mg/mL) on AA-induced ROS. (**D**) Effect of BBE and BBD on AA-induced superoxide anion radicals production. After treatment with 2.5 mM AA in the presence or absence of BBE (0.5 mg/mL) or BBD (0.5 mg/mL) for 24 h, cells were collected and incubated with 10 μM of DHE at 37 °C for 30 min, then cells were washed with PBS and evaluated by fluorescence microscope. (**E**) The quantitative data of panel (**D**) and results were expressed as mean DHE fluorescence intensity (means ± SD of three independent experiments, n = 6). (**F**) Effect of BBD (0.25, 0.5 and 1 mg/mL) on AA-induced superoxide anion radicals production *P < 0.05 represents significant compared with AA treatment group. BBE: Blackberry extract; BBD: Blackberry digests.

Increasing evidences confirmed that AA-induced toxicity was associated with intracellular excessive ROS accumulation in a dose-dependent manner^24^. Suppression of AA-induced ROS overproduction by naturally occurring antioxidants derived from fruits and vegetables is a promising strategy. Based on this, we used DCFH-DA, a specific ROS fluorescence probe, to analyze whether BBE and BBD could inhibit AA-induced intracellular ROS generation. As shown in Fig. 3A,B, exposure of AA (2.5 mM) led to a dramatic increase in fluorescence intensity (266%) in HepG2 cells compared with that in control group. Interesting, pretreatment with BBD (0.5 mg/mL) markedly scavenged AA-induced ROS and the mean fluorescence intensity decreased to 126% compared with control group. The mean fluorescence of BBE (0.5 mg/mL) treatment decreased to 223%. In addition, our study showed that BBD (ranging from 0.25, 0.5 to 1 mg/mL) pretreatment scavenged AA-induced ROS production (Fig. 3C).

Superoxide anion radical (O_2_^−^) is a particular type of ROS which can produce the highly oxidizing derivatives such as hydroxyl radical (∙OH), a very short half-life (10^−9^ s) and high reactivity radical potentially leads to most of the oxidative damage (25, 26). Hence, we investigated the superoxide anion radicals scavenging activity of BBD by a specific fluorescence probe, DHE. As illustrated in Fig. 3C,D, exposure of HepG2 cells to AA led to a high fluorescence intensity (216%) compared with that in control group. As expected, pretreatment with BBD significantly reduced fluorescence intensity to 108%. However, pretreatment with BBE slightly decreased superoxide anion radicals in HepG2 cells with no significant difference. We also observed that BBD (0.25, 0.5 to 1 mg/mL) reduced AA-induced superoxide anion radicals (Fig. 3F). It could be concluded that BBD was more effective than BBE in scavenging AA-induced intracellular ROS generation and superoxide anion radicals in HepG2 cells, suggesting that blackberry could provide protection against AA-induced toxicity through manipulating cellular redox balance.

### BBD suppressed AA-induced oxidative damage to mitochondrial membrane

**Figure 4:**
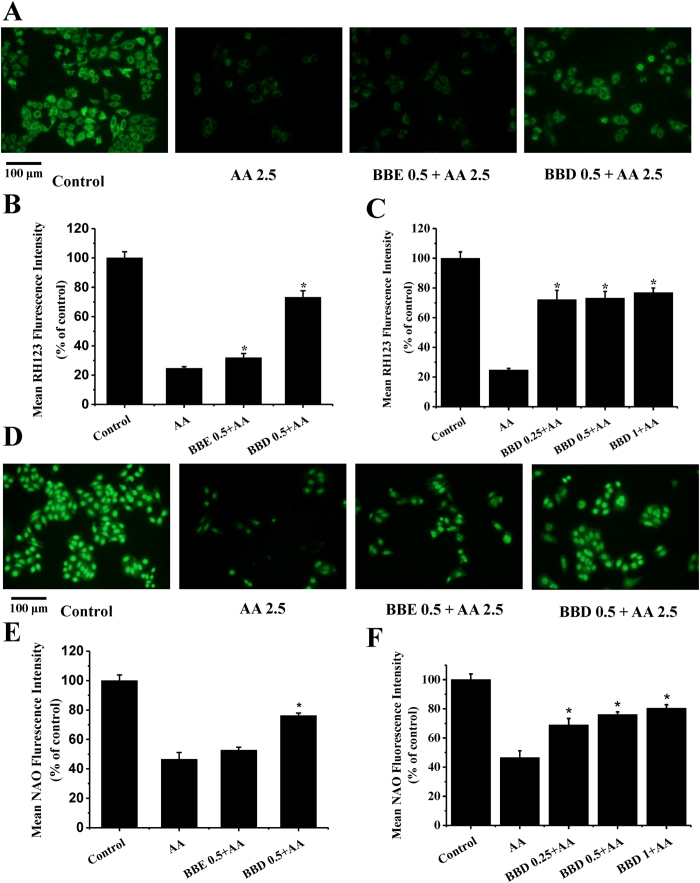
Effect of BBE and BBD on AA-induced oxidative damage to mitochondrial membrane in HepG2 cells. (**A**) Effect of BBE and BBD on MMP. After treatment with 2.5 mM AA in the presence or absence of BBE (0.5 mg/mL) or BBD (0.5 mg/mL) for 24 h, HepG2 cells were incubated with 10 μg/mL RH123 for 30 min and then adopted to fluorescence microscope analysis. (**B**) The quantitative data of panel (**A**) and results were expressed as mean RH123 fluorescence intensity (means ± SD of three independent experiments, n = 6). (**C**) Effect of BBD (0.25, 0.5 and 1 mg/mL) on MMP. (**D**) Effect of BBE and BBD on lipid peroxidation in mitochondrial membrane. After treatment with 2.5 mM AA in the presence or absence of BBE (0.5 mg/mL) or BBD (0.5 mg/mL) for 24 h. HepG2 cells were incubated with 10 μM NAO for 30 min and subsequently adopted to fluorescence microscope analysis. (**E**) The quantitative data of panel (**D**) and results were expressed as mean NAO fluorescence intensity (means ± SD of three independent experiments, n = 6). (**F**) Effect of BBD (0.25, 0.5 and 1 mg/mL) on lipid peroxidation in mitochondrial membrane. *P < 0.05 represents significant compared with AA treatment group. BBE: Blackberry extract; BBD: Blackberry digests.

Previous studies have unveiled that ROS generation was associated with mitochondrial membrane potential (MMP) collapse^25^ and cellular exposure of AA contributed to mitochondrial dysfunction^26^. On the basis of these idea, we therefore speculated that BBD also could aid to improve the integrity of mitochondrial membrane in the presence of AA. To determine the MMP levels, RH123, a specific fluorescence probe was selected in this study. As shown in Fig. 4A,B, a remarkable decrease of MMP level was observed after treatment with AA (mean fluorescence intensity is 24.6%). On the contrary, pretreatment with BBD enabled suppression of the decrease in AA-induced MMP, with the fluorescence intensity restored to 73.2% compared with that of control group. Pretreatment with BBE only increased the fluorescence intensity to 31.8% compared with that of control group. Pretreatment with BBD (0.25, 0.5 to 1 mg/mL) also improved AA-induced MMP collapse (Fig. 4C).

In addition to MMP collapse, ROS overproduction may lead to excessive lipid peroxidation in mitochondrial membrane as well^27^. Therefore, we further examined whether BBD and BBE could suppress AA-induced mitochondrial lipid peroxidation in HepG2 cells. NAO is a fluorescence probe designed for cardiolipin detection, a major mitochondrial membrane lipid component oxidized in the presence of ROS, was used to determine mitochondrial lipid peroxidation. We observed the mean fluorescence intensity significantly decreased to 46.5% of control group in the presence of AA compared with control group in the absence of AA. The value of mean fluorescence intensity increased to 76.1% of control group fluorescence intensity by pretreatment with BBD, while pretreatment with BBE slightly increased the fluorescence intensity to 52.8%. Moreover, pretreatment with BBD with increasing concentrations (ranging from 0.25, 0.5 to 1 mg/mL) improved AA-induced lipid peroxidation in mitochondrial membrane (Fig. 4F). Hereby, we confirmed that BBD was more effective in providing protection against AA-induced oxidative damage to mitochondrial membrane.

### BBD improved AA-induced GSH depletion

**Figure 5:**
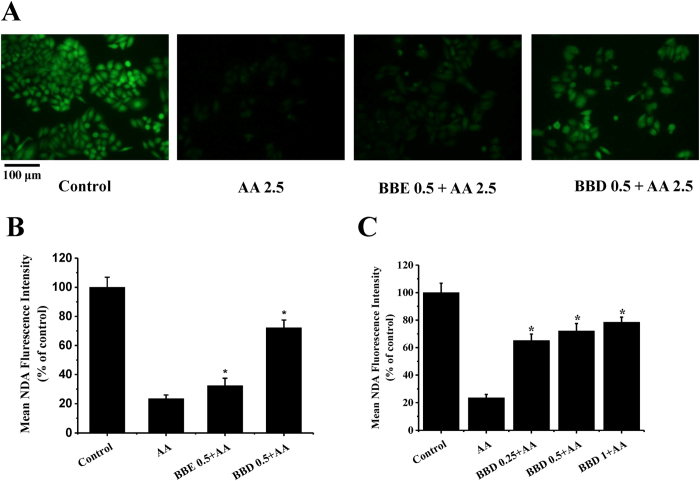
Effect of BBE and BBD on AA-induced oxidative stress to GSH antioxidant systems in HepG2 cells. (**A**) Effect of BBE and BBD on cellular GSH content. After treatment with 2.5 mM AA in the presence or absence of BBE (0.5 mg/mL) or BBD (0.5 mg/mL) for 24 h. Then cells were collected and incubated with 50 μM NDA at 37 °C for 30 min, evaluated by fluorescence microscope. (**B**) The quantitative data of panel (**A**) and results were expressed as mean NDA fluorescence intensity (means ± SD of three independent experiments, n = 6). (**C**) Effect of BBD (0.25, 0.5 and 1 mg/mL) on cellular GSH content. Values are means ± SD of three independent, *P < 0.05 represents significant compared with AA treatment group. BBE: Blackberry extract; BBD: Blackberry digests.

Glutathione (GSH) plays a critical role in neutralizing free radicals and reactive oxygen compounds^28^. Previous studies indicated that exposure to AA contributed to GSH depletion^28^. Since BBD exhibited a profound effect in scavenging AA-induced intracellular ROS and ameliorating oxidative damage to mitochondrial membrane, we postulated that BBD also could attenuate AA-induced GSH depletion in HepG2 cells. To determine the intracellular GSH levels, NDA, a highly selective fluorescence probe, was used for GSH detection. In the present study, we noticed that AA (2.5 mM) led to a marked decrease (23.5% of control group) in GSH content compared with control group (Fig. 5A,B). Noticeably, pretreatment with BBD (0.5 mg/mL) attenuated AA-induced GSH depletion and the fluorescence intensity was increased to 72.2% compared with that of control group. In line with our aforementioned results, BBE (0.5 mg/mL) was less efficacious than BBD (0.5 mg/mL) in improving AA-induced GSH depletion. Further study indicated that pretreatment of BBD (0.25, 0.5 and 1 mg/mL) also restored GSH contents (Fig. 5C). These observations implied that BBD might provide protection against AA-induced oxidative damage in HepG2 cells via regulating GSH antioxidant systems to cope with oxidative stress.

### BBD improved the SOD and CAT enzyme activity

**Figure 6:**
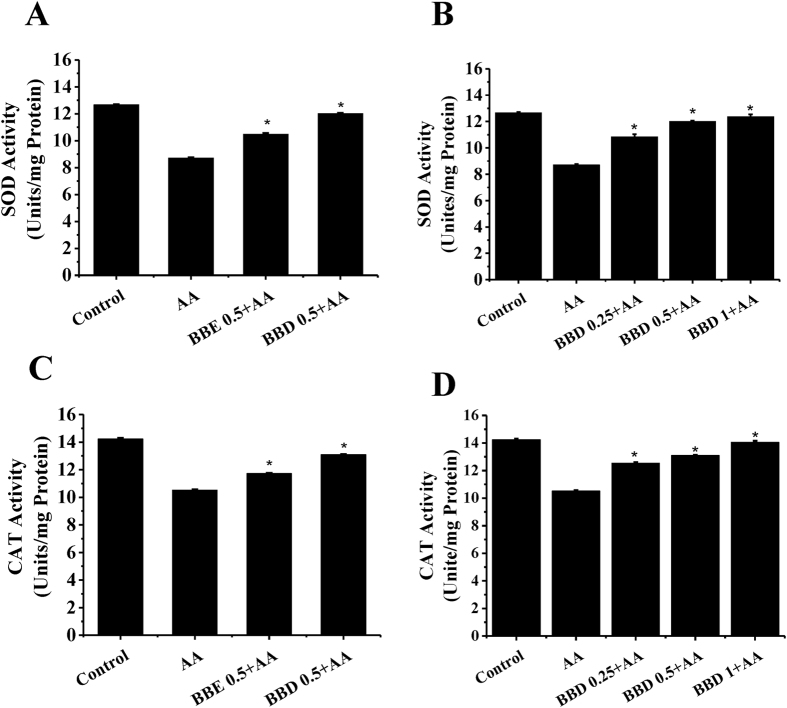
Effect of BBE and BBD on SOD and CAT in HepG2 cells. (**A**) Effect of BBE (0.5 mg/mL) and BBD (0.5 mg/mL) on SOD activity in the presence of AA. SOD activity was measured and expressed as U/mg protein. (**B**) Effect of BBD (0.25, 0.5 and 1 mg/mL) on SOD activity in the presence of AA. (**C**) Effect of BBE (0.5 mg/mL) and BBD (0.5 mg/mL) on CAT activity in the presence of AA. CAT activity was measured and expressed as U/mg protein. (**D**) Effect of BBD (0.25, 0.5 and 1 mg/mL) on CAT activity in the presence of AA. Values are means ± SD of three independent, ^*^P < 0.05 represents significant difference between conditions. BBE: Blackberry extract; BBD: Blackberry digests. SOD: superoxide dismutase; CAT: catalase.

Superoxide dismutase (SOD) and Catalase (CAT) play a crucial role in maintaining cellular redox homeostasis^29^,^30^. Thus, we next determined the effect of BBE and BBD on the enzyme activities of SOD and CAT. As shown in Fig. 6A,C, after treatment with 2.5 mM AA for 24 h, the enzyme activity of SOD was decreased to 68.8% of control group. For CAT activity, similar change was found, and the enzyme activity was decreased to 82.9% of control group. Both BBE and BBD pretreatment significantly improved the enzyme activities of SOD and CAT in the presence of AA. Pretreatment with 0.5 mg/mL BBD significantly increased the activity of SOD and CAT to 11.72 U/mg (82.9% of control group) and 13.1 U/mg (92.6% of control group) in the presence of AA, respectively. We also performed a dose-response determination and observed a further slight increase in the enzyme activities of SOD and CAT with increasing concentrations of BBD (ranging from 0.25, 0.5 and 1 mg/mL) (Fig. 6B,D). Functional components undergo some structural modifications and functional alteration during GI digestion as a result of GI tract movement, effects of digestive enzymes, and many digestive substances secreted from viscera and gland^31^. Previous studies indicated that cellular antioxidant activity was enhanced after *in vitro* digestion^32^, which was similar to our study.

Our preliminary data showed that GI-digested blackberry enabled to inhibit AA-induced ROS accumulation. Further observations indicated that blackberry extract prevented MMP decrease and mitochondrial membrane oxidation, thereby reducing the oxidative damage to mitochondrial membrane. GSH is one of the major cellular antioxidants within cells that is responsible for maintaining a critical tight control of redox status balance^33^. Our previous study indicated that GSH content depleted in Caco-2 cells if exposed to AA in dose-dependent^15^. Likewise, in this study, based on the observations, blackberry serves as a good ROS scavenger. The following research revealed that both bioactivities of SOD and CAT were heavily enhanced by pretreatment of BBD. As a consequence, blackberry extract provides protection against AA-induced oxidative damage in HepG2 cells.

### Identification of phenolic compounds from BBD

Since *in vivo* transport and metabolic mechanisms are complex and cannot be easily reproduced, *In vitro* studies may provide a relatively simple but effective approach to predict the bioaccessibility and bioavailability of some phytochemicals under simulated gastrointestinal conditions.

**Figure 7:**
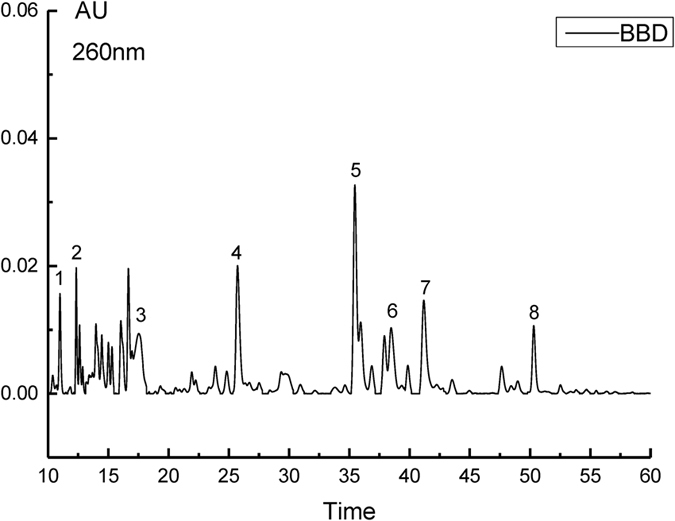
The HPLC chromatogram of blackberry after GI digestion detected at 260 nm.

According to our results, blackberry digests (BBD) afforded a better protection against AA-induced oxidative damage. Biological activities of BBD were highly associated with their phenolic compounds. However, the phenolic composition of BBD are not well established so far. Therefore, high resolution LC/MS/MS was used to identify phenolic compounds in blackberry digests. The results were displayed in Fig. 7 and summarized in Table 1. As shown in Fig. 7, eight phenolic compounds were identified in blackberry digests according to their MS/MS information (Supplemental Figs 1–8) and published data. It can be found that ellagic acid and its derivatives, such as ellagic acid pentoside, ellagic acid glucuronoside and methyl-ellagic acid pentoside, are major phenolic compounds in blackberry digests. The mass unit of 300.9991 ([M - H]^−^) has been previously identified as ellagic acid^34^. The loss of 15 mass units (315.0150–300.9953), 132 mass units (433.0405–300.9993) and 176 mass uints (491.0460–315.0150) could be considered to methyl, pentose and glucuronoside, respectively^35^. Moreover, cyanidin glucoside, gallic acid and vanillic acid hexoside were also identified in blackberry digests based on their MS information. These phenolic compounds, especially ellagic acid and cyanidin glucoside, have been proved to possess potent antioxidant activity. Therefore, the protective effect of BD against AA-induced oxidative damage may be related to these phenolic compounds.

## Conclusions

In summary, this study revealed that GI digestion increased the ability of blackberry extract to inhibit AA-induced oxidative stress in HepG2 cells by diminishing intracellular ROS, ameliorating mitochondrial integrity and preventing GSH depletion. Further investigation showed the activities of both SOD and CAT were significantly increased after pretreatment of BBD. In addition, LC/MS/MS analysis identified eight phenolic compounds with high contents in BBD as primary active compounds responsible for antioxidant action. Together, our study revealed that blackberry underwent gastrointestinal digestion improved the protective activity that resulted in a better performance in ameliorating AA-induced oxidative damage, which might provide an alternative perspective on health-promoting effect of blackberry in preventing exogenous pollutant-induced oxidative stress.

## Methods

### Chemicals and reagents

6-hydroxy-2,5,7,8-tetramethylchroman-2-carboxylic acid (Trolox), 3-(4,5-dimthyl-2-thiazolyl)-2,5-diphenyl-2-H-tetrazolium bromide (MTT), Nonyl Acridine Orange (NAO), dihydroethidium (DHE), Hochest 33258, Folin & Ciocalteu’s phenol reagent, pepsin, pancreatin, and bile salts were purchased from the Sigma-Aldrich (St. Louis, MO, USA). Dichlorodihydrofluorescein diacetate (DCFH-DA), Rhodamine 123 (RH123), Naphthalene-2,3-dicarboxal-dehyde (NDA) were purchased from Molecular Probes, Inc. (Eugene, OR, USA). Trizol was purchased from Life Technologies (Carlsbad, CA, USA). Cell lysis buffer, BCA Protein assay kit, Catalase assay kit, and total superoxide dismutase assay kit were obtained from Beyotime Institute of Biotechnology., Ltd (Shanghai, China). All other reagents used were of analytical grade.

### Blackberries

Blackberry fruits were gathered from Nanjing in Jiangsu Province, China. Fresh fruits were washed thoroughly with sterile distilled water and were dried under shade in a clean, dust free environment. The fruits were screened and uniformed based on the size, weight, shape and moisture content.

### Gastrointestinal (GI) digestion

Blackberry underwent *in vitro* GI digestion process as previously described with slight modifications^19^,^36^. Briefly, 20 mL of water was added to 20 g of fresh blackberry fruit and homogenized in a commercial homogenizer. After that, 20 g of homogenate was acidified to pH 2 by the addition of 5 M HCl, and then porcine pepsin (6000 units) was added and incubated at 37 °C in a shaking water bath for 1.5 h at 15× g. The pH was subsequently adjusted to 6.5 using 1 M sodium bicarbonate, followed by adding 5 mL of pancreatin (containing 4 mg/mL trypsin and 25 mg/mL porcine bile salts). Then this solution was incubated at 37 °C in a shaking water bath for 2 h at 15× g. Finally, the mixture was diluted to a volume of 25 mL with distilled water and centrifuged at 4193× g for 6 min. Supernatant was collected, named as blackberry digest (BBD). Blackberry extract sample before GI digestion (BBE) was prepared as follows. 20 g of fresh blackberry fruit was homogenized and diluted to a final volume of 25 mL. All BBE and BBD stock solutions were sealed and stored at −20 °C for further investigation. The blank (without adding the blackberry samples) was also incubated under the same conditions and used for the correction of interferences from the digestive fluids.

### Cell viability assay

Cell viability was tested by the MTT method as previously described. Briefly, HepG2 cells were seeded into 96-well microtiter plates at a concentration of 4 × 10^3^ cells/well. AA was diluted in serum-free culture medium (2.5 mM). After incubation for 24 h, cells were treated with 2.5 mM AA in the presence or absence of BBE or BBD. After 24 h, cells were incubated with 0.5 mg/mL MTT for 4 h. A formation of formazan precipitate was observed and was dissolved into 150 μL of Dimethyl sulfoxide (DMSO). The absorbance of mixture was detected at 490 nm by microplate reader (Tecan Infinite M200) in triplicate for each test.

### Determination of cellular reactive oxygen species (ROS) and superoxide anion radicals

Cellular ROS was monitored according to a previously described method with slight modifications^37^. Briefly, HepG2 cells were seeded into 12-well microtiter plates at a concentration of 5 × 10^4^ cells/well for 24 h incubation. HepG2 cells were then treated with 2.5 mM AA in the presence or absence of BBE or BBD for 24 h, followed by incubating with 10 μM DCFH-DA at 37 °C. After 30 min incubation with the fluorescence probe, cells were washed with PBS and then evaluated immediately under fluorescence microscope. The results were expressed as mean DCF fluorescence intensity calculated by image analysis software ImageProPlus 6.0 (Media Cybernetics, Inc.). Cellular superoxide anion radicals was analyzed as previously described^38^. Briefly, HepG2 cells were treated with 2.5 mM AA in the presence or absence of BBE or BBD for 24 h, followed by incubating with 50 μg/mL DHE at 37 °C for 30 min. Cells were washed with PBS after incubation with the fluorescence probe and then evaluated immediately by fluorescence microscope. The results were expressed as mean DHE fluorescence intensity.

### Determination of cellular mitochondrial membrane potential (MMP) and mitochondrial membrane lipid oxidation

MMP assay was determined according to the approach as previously described with slight modifications^37^. Briefly, HepG2 cells were treated with 2.5 mM AA in the presence or absence of BBE or BBD for 24 h, followed by incubating with 10 μg/mL RH123 at 37 °C for 30 min. Cells were washed with PBS after incubation with the fluorescence probe and then evaluated immediately by fluorescence microscope. The results were expressed as mean RH123 fluorescence intensity. Cellular lipid oxidation was determined according to the procedure as previously described^38^. Briefly, HepG2 cells were treated with 2.5 mM AA in the presence or absence of BBE or BBD for 24 h, followed by incubating with 50 μg/mL NAO at 37 °C for 30 min. Cells were washed with PBS after incubation with the fluorescence probe and then evaluated immediately by fluorescence microscope. The results were expressed as mean NAO fluorescence intensity.

### Determination of cellular glutathione (GSH)

Cellular GSH was carried out according to the procedure as previously described with some modifications^38^. Briefly, HepG2 cells were treated with 2.5 mM AA in the presence or absence of BBE or BBD for 24 h, followed by incubating with 50 μg/mL NDA at 37 °C for 30 min. Cells were washed with PBS after incubation with the fluorescence probe and then evaluated immediately by fluorescence microscope. The results were expressed as mean NDA fluorescence intensity.

### Hoechst 33258 assay

The DNA-bound Hoechst 33258 assay was conducted as previously described^39^. Briefly, after treatment, cells were incubated with 10 μg/mL Hoechst 33258 at 37 °C for 30 min. Cells were washed with PBS after incubation with the fluorescence probe and then evaluated immediately by fluorescence microscope.

### Determination of intracellular superoxide dismutase (SOD) and catalase (CAT) activity

Total SOD activities of the samples was determined using the Total Superoxide Dismutase Assay Kit with WST-8 and Total CAT activities of the samples was determined using the Catalase Assay Kit (Beyotime Institute of Biotechnology, Jiangsu, China) based on the protocols provided by the manufacturer.

### HPLC/MS/MS analysis

The experiment was carried out on Waters UPLC system equipped with Promosil C18 column (4.6 × 250 mm, 5 μm) and Triple-TOF Mass Spectrometry System (AB SCIEX, Triple-TOF 5600plus Framingham, USA). The solvents were acetonitrile (A) and deionized water (B), both containing 0.1% formic acid. Samples (before and after GI digestion) were filtered through 0.45 μm membrane and then separated using the following linear gradient: from 5% to 8% B in 4 min, from 8% to 12.8% B in 4 min, from 12.8% to 20% B in 32 min, from 20% to 28% B in 15 min, from 28% to 64% B in 12 min, isocratic elution in 5 min, from 64% to 80% in 3 min and then equilibrated in 10 min, with an injection volume of 10 μL and a flow rate of 0.5 mL/min. The optimal MS conditions were listed as follows: the scan range (m/z) was from 100 to 1500, detection performed in negative ion mode, with a source temperature and voltage at 550 °C and 4.5 KV respectively, the UV detector set at 260 nm. Identification was based on the ion molecular mass, MS^2^ and UV-visible spectra. The content of the identical compound in blackberry extracts (before and after GI digestion) was compared using peak area. The contents of anthocyanins identified were achieved by calculating the percent area of individual peaks of all peaks of chromatogram obtained at 520 nm and cyanidin-3-O-glucoside was used as standard.

### Statistical analysis

All data were expressed as means ± standard deviations (SD). Data and statistical analyses were analyzed by t-test or one-way ANOVA using SPSS (version19.0), *p < 0.05* was considered statistically significant.

## Additional Information

**How to cite this article**: Chen, W. *et al. In vitro* gastrointestinal digestion promotes the protective effect of blackberry extract against acrylamide-induced oxidative stress. *Sci. Rep.* **7**, 40514; doi: 10.1038/srep40514 (2017).

**Publisher's note:** Springer Nature remains neutral with regard to jurisdictional claims in published maps and institutional affiliations.

## Supplementary Material

Supplemental Material

## Figures and Tables

**Table 1:** Identification of phenolic compounds in blackberry digests.

peak	Rt (min)	MS	MS^2^	Tentative identification
1	10.98	169.0155	125.0239	Gallic acid
2	12.61	329.0877	167.0345/152.0104	Vanillic acid hexoside
3	17.54	447.0931	285.0399	Cyanidin glucoside
4	25.72	477.0307	301.0001	Ellagic acid glucuronoside
5	35.56	433.0405	300.9990	Ellagic acid pentoside
6	38.57	491.0460	315.0150/300.9953	Methyl-ellagic acid glucuronoside
7	41.26	300.9991	283.9961	Ellagic acid
8	50.31	447.0556	315.0145/300.9958	Methyl-ellagic acid pentoside
