# Supplementary material for: In vitro gastrointestinal digestion promotes the protective effect of blackberry extract against acrylamide-induced oxidative stress
Source: Sci Rep. 2017 Jan 13;7:40514. doi: 10.1038/srep40514 (PMC5233992; doi:10.1038/srep40514)
Supplement: Supplemental Material [file srep40514-s1.pdf]

In vitro gastrointestinal digestion promotes the protective effect of blackberry extract against acrylamide-induced oxidative stress

Wei Chen*, Hongming Su, Yang Xu, Chao Jin

Department of Food Science and Nutrition, Zhejiang University, Hangzhou 310058, China.

* Correspondence and requests for materials should be addressed to W.C. (email: zjuchenwei@zju.edu.cn).

This supplementary file includes Supplementary Figures 1-8.

Fig. S1

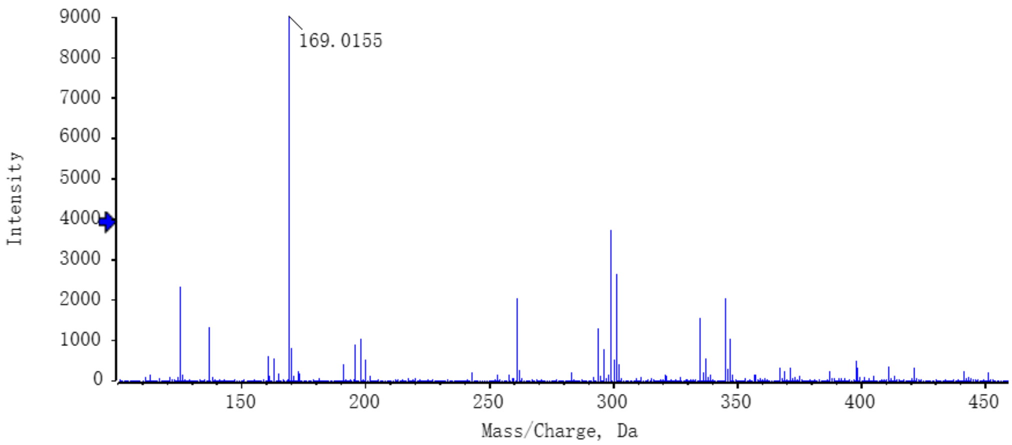

Mass spectra of gallic acid (m/z 169)

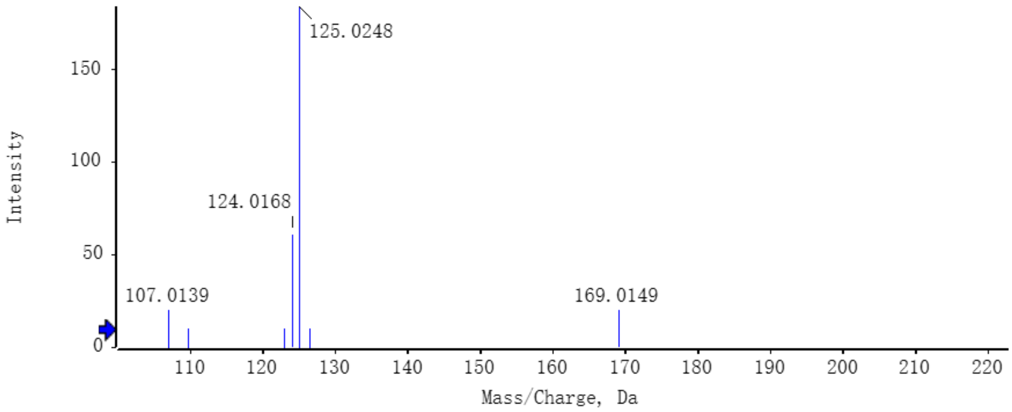

MS/MS of gallic acid (m/z 169)

Supplementary Figure 1. Mass spectra of gallic acid (m/z 169) and MS/MS of gallic acid (m/z 169)..

Fig. S2

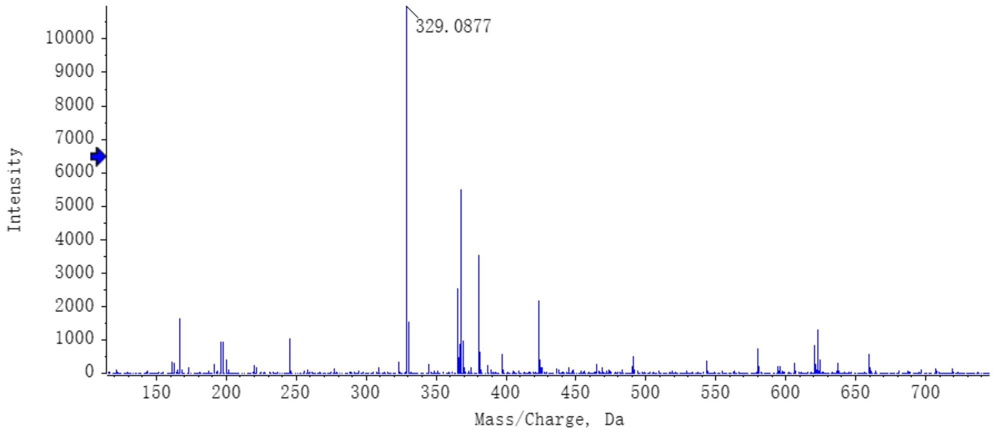

Mass spectra of vanillic acid hexoside (m/z 329)

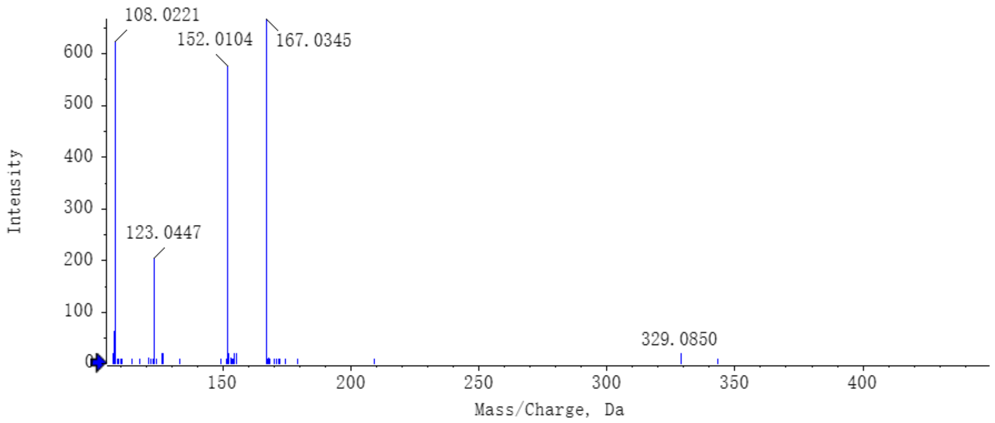

MS/MS of vanillic acid hexoside (m/z 329)

Supplementary Figure 2. Mass spectra of vanillic acid hexoside (m/z 329) and MS/MS of vanillic acid hexoside (m/z 329).

Fig. S3

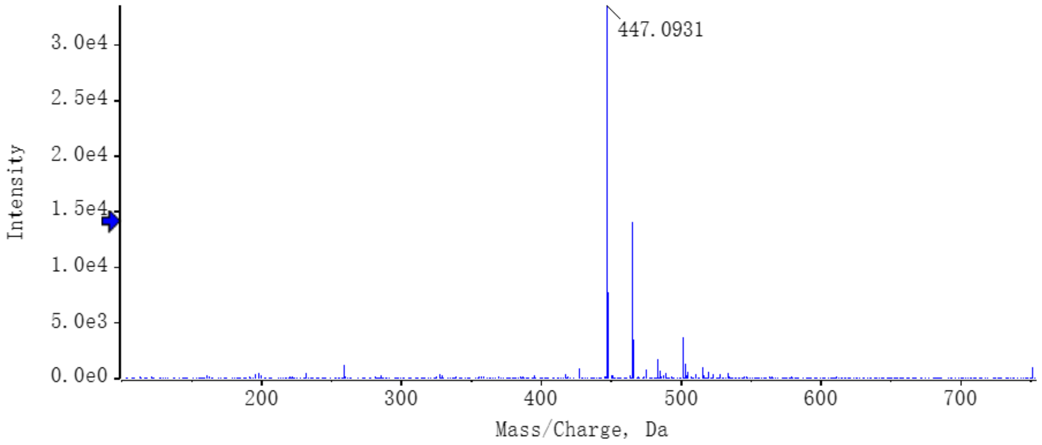

Mass spectra of cyanidin glucoside (m/z 447)

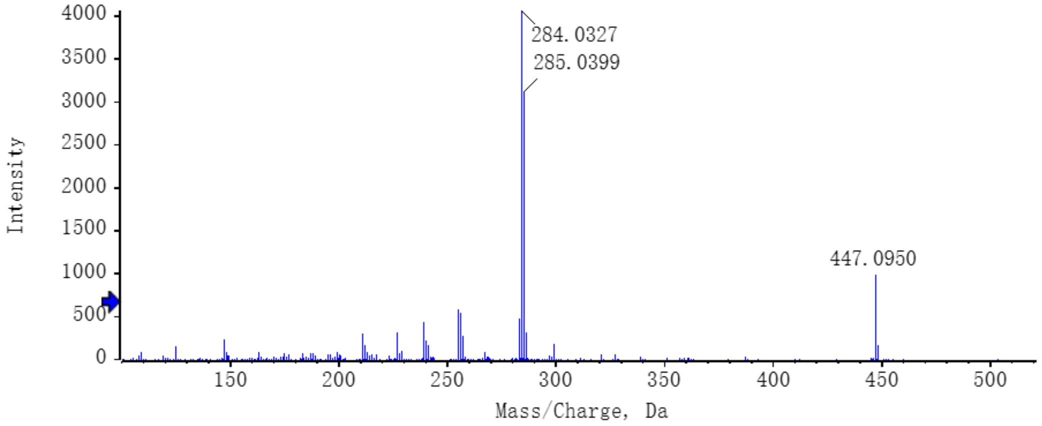

MS/MS of cyanidin glucoside (m/z 447)

Supplementary Figure 3. Mass spectra of cyanidin glucoside (m/z 447) and MS/MS of cyanidin glucoside (m/z 447).

Fig. S4

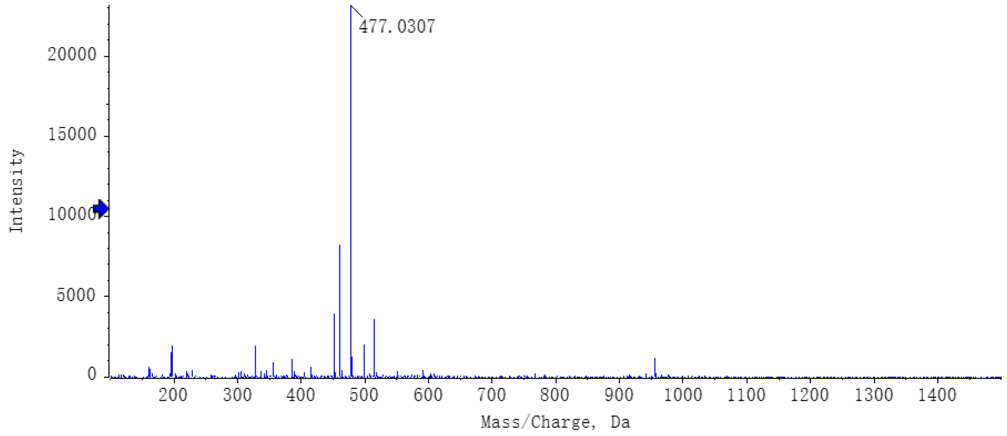

Mass spectra of ellagic acid glucuronoside (m/z 477)

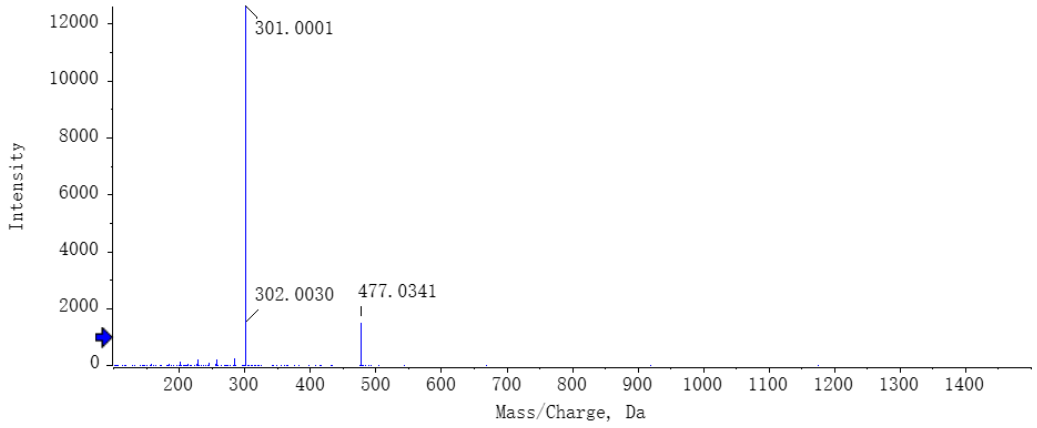

MS/MS of ellagic acid glucuronoside (m/z 477)

Supplementary Figure 4. Mass spectra of ellagic acid glucuronoside (m/z 477) and MS/MS of ellagic acid glucuronoside (m/z 477).

Fig. S5

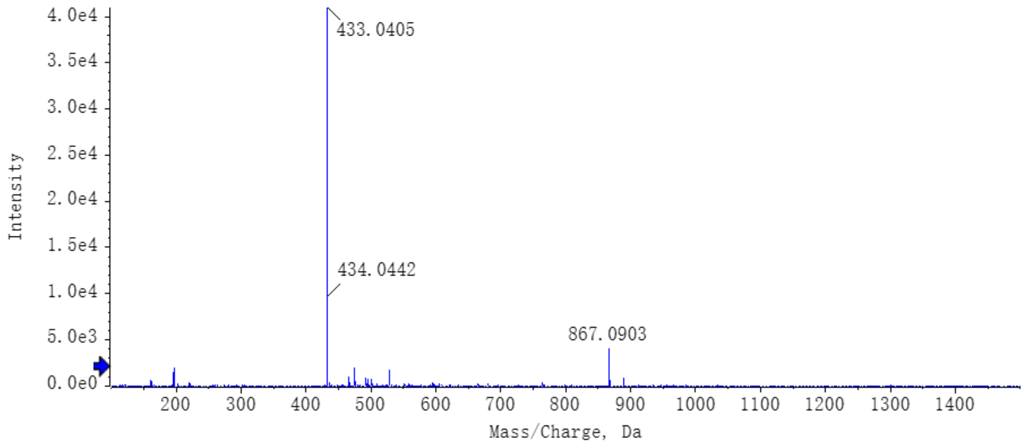

Mass spectra of ellagic acid pentoside (m/z 433)

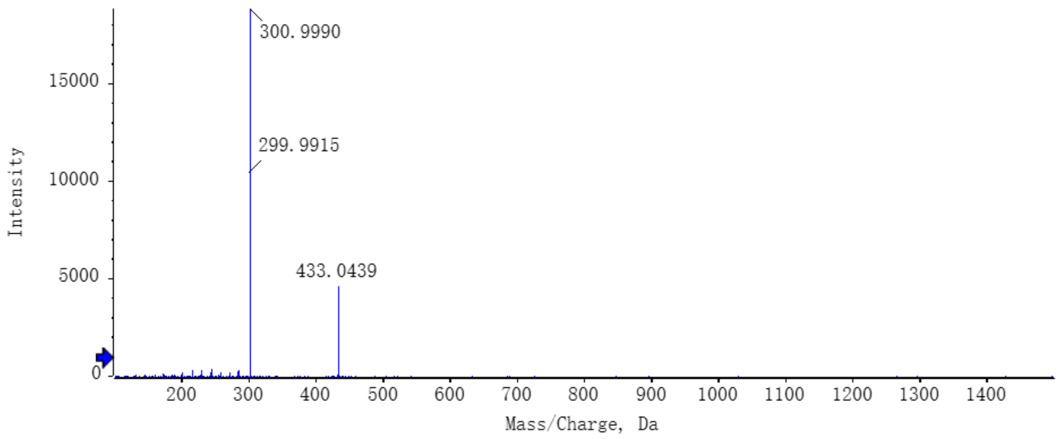

MS/MS of ellagic acid pentoside (m/z 433)

Supplementary Figure 5. Mass spectra of ellagic acid pentoside (m/z 433) and MS/MS of ellagic acid pentoside (m/z 433).

Fig. S6

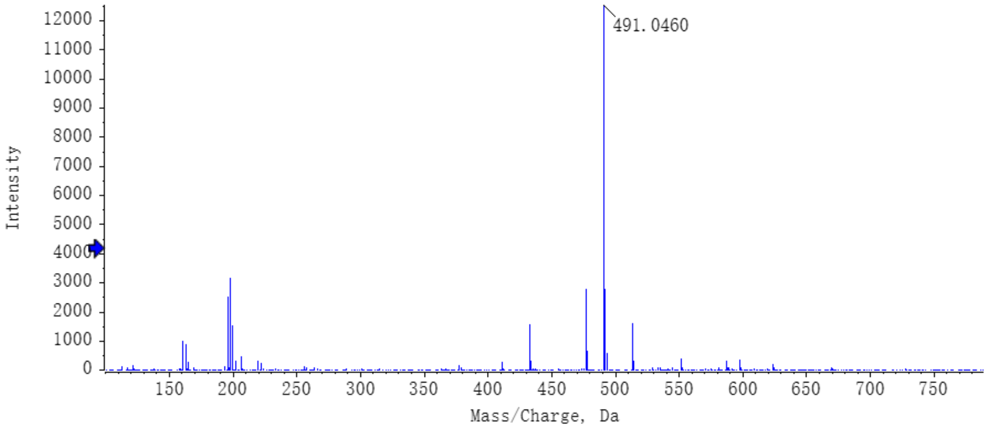

Mass spectra of Methyl-ellagic acid glucuronoside (m/z 491)

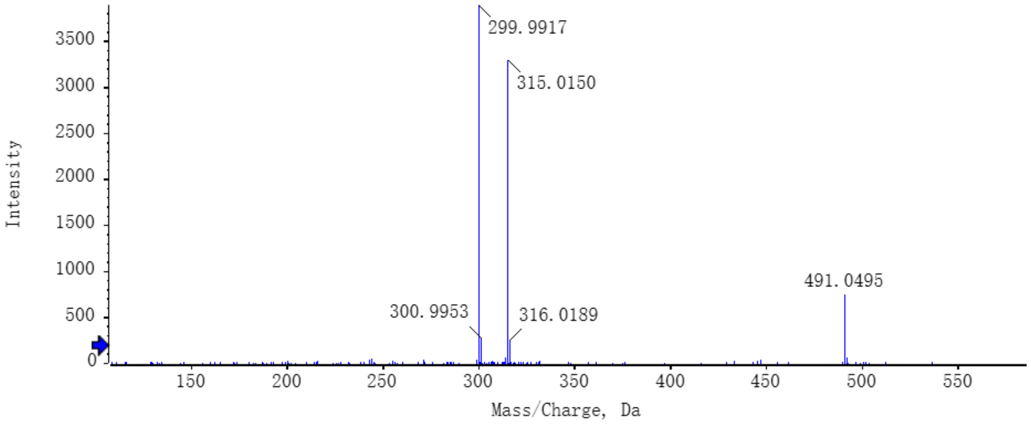

MS/MS of Methyl-ellagic acid glucuronoside (m/z 491)

Supplementary Figure 6. Mass spectra of Methyl-ellagic acid glucuronoside (m/z 491) and MS/MS of Methyl-ellagic acid glucuronoside (m/z 491).

Fig. S7

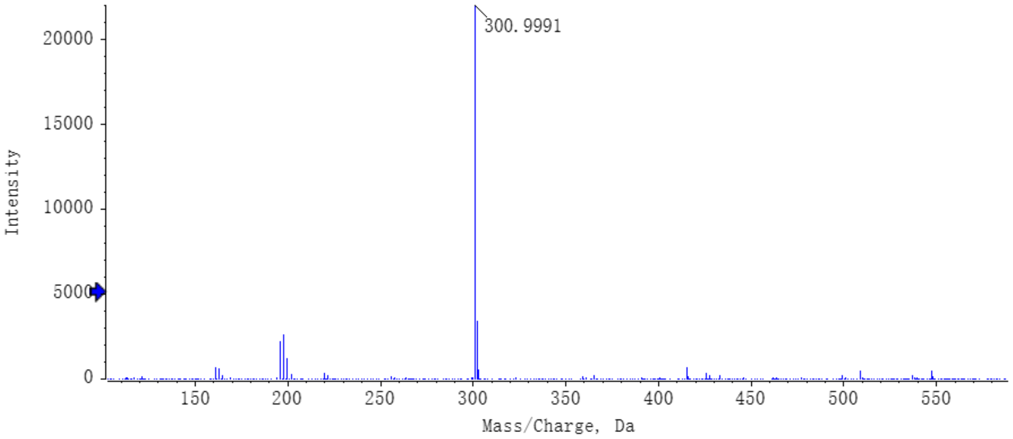

Mass spectra of ellagic acid (m/z 301)

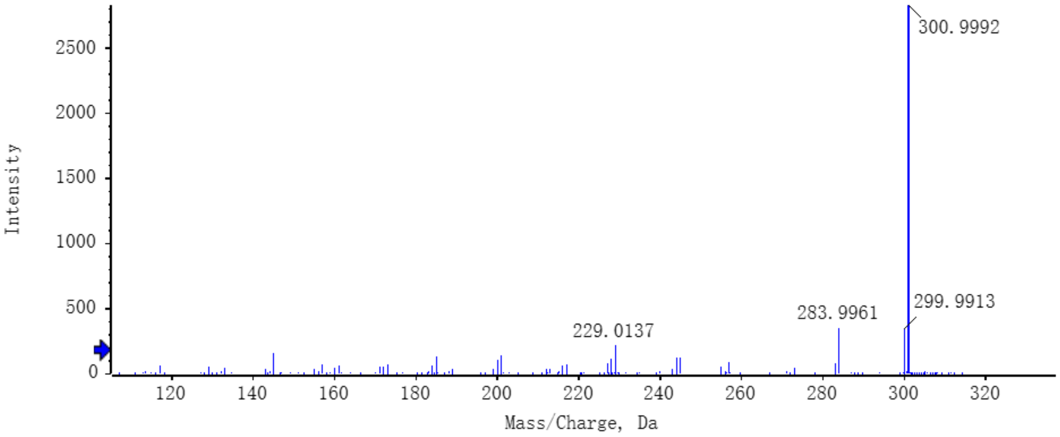

MS/MS of ellagic acid (m/z 301)

Supplementary Figure 7. Mass spectra of ellagic acid (m/z 301) and MS/MS of ellagic acid (m/z 301).

Fig. S8

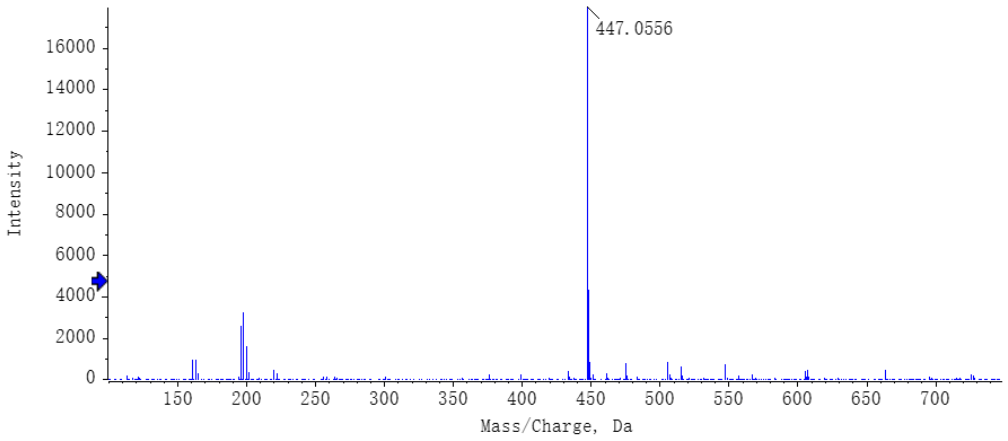

Mass spectra of methyl-ellagic acid pentoside (m/z 447)

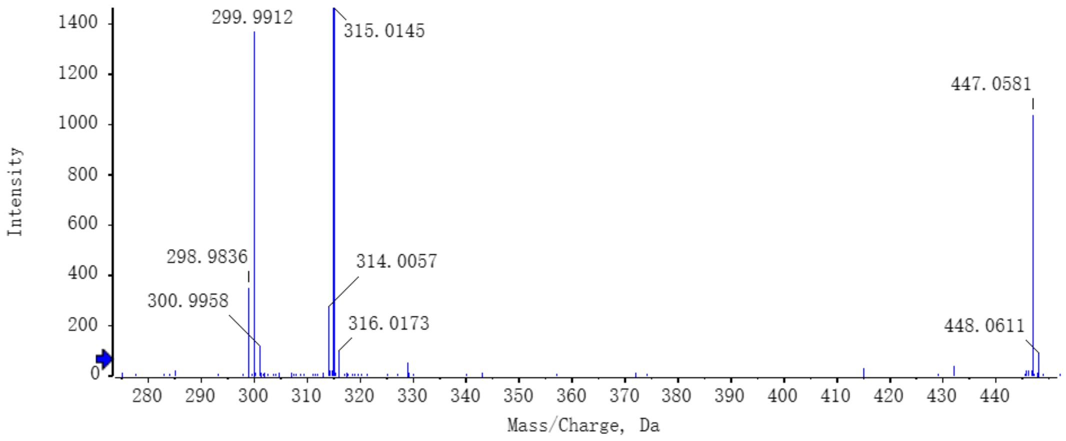

MS/MS of methyl-ellagic acid pentoside (m/z 447)

Supplementary Figure 8. Mass spectra of methyl-ellagic acid pentoside (m/z 447) and MS/MS of methyl-ellagic acid pentoside (m/z 447).
